# Clinical Characteristics of Diabetic Cardiovascular Autonomic Neuropathy in Korean Individuals With Type 2 Diabetes

**DOI:** 10.1111/1753-0407.70245

**Published:** 2026-07-10

**Authors:** Seon Mee Kang, Kyong Hye Joung, Chong Hwa Kim, Jae‐Seung Yun, Jong Chul Won, Jae Hyuk Lee, Chang Won Lee, Hyuk‐Sang Kwon, Ie Byung Park, Tae Sun Park

**Affiliations:** ^1^ Division of Endocrinology and Metabolism, Department of Internal Medicine Soonchunhyang University College of Medicine Cheonan Republic of Korea; ^2^ Division of Endocrinology and Metabolism, Department of Internal Medicine Chungnam National University Sejong Hospital Chungnam National University School of Medicine Sejong Republic of Korea; ^3^ Division of Endocrinology and Metabolism, Department of Internal Medicine Sejong General Hospital Bucheon Republic of Korea; ^4^ Division of Endocrinology and Metabolism, Department of Internal Medicine, St. Vincent's Hospital College of Medicine, the Catholic University of Korea Suwon Republic of Korea; ^5^ Division of Endocrinology and Metabolism Department of Internal Medicine, Gimpo Woori Hospital Gimpo Republic of Korea; ^6^ Division of Endocrinology and Metabolism, Department of Internal Medicine, Myongji Hospital Hanyang University Goyang Republic of Korea; ^7^ Division of Endocrinology and Metabolism, Department of Internal Medicine Busan St. Mary's Hospital Busan Republic of Korea; ^8^ Division of Endocrinology and Metabolism, Department of Internal Medicine, Yeouido St. Mary's Hospital College of Medicine, the Catholic University of Korea Seoul Republic of Korea; ^9^ Division of Endocrinology and Metabolism, Department of Internal Medicine Gachon University Gil Medical Center Incheon Republic of Korea; ^10^ Division of Endocrinology and Metabolism, Department of Internal Medicine Jeonju Hospital Jeonju Republic of Korea

**Keywords:** associated factors, cardiovascular autonomic neuropathy, heart rate variability, Korea, QTc interval, subgroup analysis, type 2 diabetes mellitus

## Abstract

**Aim:**

This study aimed to evaluate the prevalence, clinical characteristics, and factors associated with cardiovascular autonomic neuropathy (CAN) in Korean individuals with long‐standing type 2 diabetes mellitus (T2DM).

**Methods:**

A total of 876 participants with long‐standing T2DM were enrolled in this multicenter cross‐sectional study. CAN was evaluated using standard cardiovascular autonomic reflex tests (CARTs) and corrected QT interval (QTc), with additional heart rate variability (HRV) analysis. CAN severity was classified as early, definite, or severe. Multivariable logistic regression analysis was performed to identify factors independently associated with CAN.

**Results:**

CAN was detected in 88.4% of participants. Individuals with CAN showed reduced HRV indices and prolonged QTc intervals. Multivariable analysis revealed advanced age (OR = 1.062; 95% CI: 1.035–1.091; *p* < 0.0001) and higher systolic blood pressure (OR = 1.036; 95% CI: 1.022–1.051; *p* < 0.0001) as factors independently associated with CAN.

**Conclusion:**

CAN is highly prevalent among Korean individuals with long‐standing T2DM. Older age and elevated systolic blood pressure were independently associated with CAN, highlighting the importance of routine CAN screening and careful blood pressure management.

## Highlights

- The prevalence of CAN was remarkably high (88.4%) in Korean individuals with long‐standing T2DM.
- Affected individuals exhibited reduced HRV and prolonged QTc intervals, reflecting significant autonomic dysfunction.
- Older age and higher systolic blood pressure were identified as factors independently associated with CAN.

## Graphical abstract

In long‐standing type 2 diabetes, cardiovascular autonomic neuropathy (CAN) was highly prevalent (88.4%), characterized by reduced heart rate variability (HRV) and prolonged QTc intervals. Advanced age and higher systolic pressure predicted CAN, indicating the importance of routine autonomic assessment and early blood pressure management, even with adequate glycemic control to reduce cardiovascular risk.

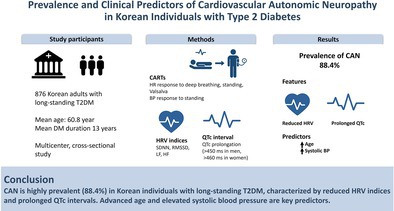

## 1 Introduction

Diabetic cardiovascular autonomic neuropathy (CAN) is a common but frequently overlooked and inadequately characterized complication of diabetes mellitus [1]. CAN is characterized by diminished autonomic regulation of the cardiovascular system, arising from damage to autonomic nerve fibers supplying the heart and blood vessels. This dysfunction results in impaired control of heart rate and disturbances in vascular tone [2]. CAN is linked to a markedly elevated risk of silent myocardial ischemia, arrhythmias, and sudden cardiac death, thereby significantly increasing morbidity and mortality in diabetic patients [3]. Although the precise pathophysiological mechanisms underlying CAN are not fully elucidated, hyperglycemia is identified as the primary factor in CAN development. Other metabolic and cardiovascular risk factors—such as insulin resistance, dyslipidemia, obesity, hypertension, and systemic inflammation—play a crucial role in the pathogenesis of CAN, especially among individuals with type 2 diabetes mellitus (T2DM) [4, 5].

For diagnosing CAN, conventional cardiovascular autonomic reflex tests (CARTs) remain the benchmark method [6]. However, additional techniques such as heart rate variability (HRV) analysis and electrocardiographic indicators, including corrected QT (QTc) interval prolongation, are increasingly being adopted in clinical practice [2]. Despite the availability of these diagnostic approaches, CAN commonly advances without obvious symptoms in its early stages. When symptoms become apparent, they are usually vague and nonspecific, impeding timely detection and frequently indicating that the disease is already at an advanced stage [7]. Additionally, the lack of universally accepted diagnostic criteria remains a significant barrier to both clinical identification and epidemiological investigations of CAN.

The reported prevalence of CAN demonstrates substantial variability depending on the population under investigation and the diagnostic techniques utilized. Several studies have documented prevalence rates ranging from 29% to 54% in individuals with type 1 diabetes, 12% to 73% in those with type 2 diabetes, and 5.9% to 11.4% even among individuals with prediabetes [5]. Nevertheless, comprehensive data on the prevalence of CAN specifically in the Korean population are still scarce. Recently, Lee et al. provided the prevalence and clinical characteristics of CAN in Korean individuals with long‐standing diabetes [8]. However, as their study encompassed both type 1 and type 2 diabetes, the findings may not accurately reflect the characteristics of the predominant T2DM population in Korea. Notably, national data show that among young‐onset diabetes cases (aged 20–40 years), the proportion of T2DM increased significantly from 93.3% to 97.2% during 2006 to 2015 [9].

Accordingly, this multicenter cross‐sectional study was designed to assess the prevalence of CAN in Korean patients with long‐standing T2DM using standardized CARTs, HRV indices, and QTc interval assessments. Furthermore, we aimed to determine the clinical characteristics and factors independently associated with CAN in this demographic.

## 2 Materials and Methods

### 2.1 Study Design and Participants

This investigation is a retrospective, multicenter, cross‐sectional subanalysis of previously collected data from individuals with T2DM recruited from eight university‐affiliated and general hospitals across Korea [8]. Inclusion criteria specified patients with confirmed T2DM and a minimum disease duration of 10 years. Subjects with a diagnosis of type 1 diabetes mellitus (T1DM) or secondary diabetes mellitus were specifically excluded. The analysis further excluded those with acute infections, liver cirrhosis, thyroid disease, vitamin B12 deficiency, malignancy, current use of anti‐arrhythmic drugs, pacemaker implantation, hypoglycemia (glucose < 70 mg/dL), severe fasting hyperglycemia (glucose ≥ 200 mg/dL), or advanced age (≥ 80 years).

### 2.2 Data Collection Clinical Measurements

Electronic medical records were reviewed retrospectively for patients who were assessed for CAN between January 1, 2015, and June 30, 2018. Clinical variables, such as age, gender, diabetes duration, anthropometric measurements, blood pressure, smoking status, alcohol use, medical history, and medication profiles were collected. Laboratory assessments included fasting glucose, glycated hemoglobin (HbA1c), fasting and postprandial c‐peptide, serum creatinine, lipid profiles (total cholesterol, high‐density lipoprotein cholesterol, and low‐density lipoprotein cholesterol, and triglyceride), and urine albumin‐to‐creatinine ratio (UACR). Diabetic complications, including retinopathy, nephropathy, and peripheral neuropathy, were identified using comprehensive ophthalmologic examinations, UACR testing, and neurologic evaluations.

### 2.3 Assessment of Cardiovascular Autonomic Neuropathy

CAN was evaluated using a standardized set of autonomic function tests [10] performed with the DiCAN system (Medicore Co. Ltd., Seoul, Korea), which consisted of the following assessments:

- Heart Rate Variability (HRV): Assessed via 5‐min electrocardiogram (ECG) recordings. Analyses included both time‐domain parameters (standard deviation of normal‐to‐normal intervals [SDNN], root mean square of successive differences [RMSSD]) and frequency domain indices (low‐frequency [LF], high‐frequency [HF], and total power [TP]).
- Blood Pressure Response to Postural Change: Orthostatic hypotension was defined as a decrease in systolic blood pressure (SBP) ≥ 20 mmHg or a decrease in diastolic blood pressure (DBP) ≥ 10 mmHg upon standing.
- Valsalva Maneuver: Evaluated as the ratio between the longest RR interval after the maneuver and the shortest RR interval during the maneuver, with a ratio < 1.20 considered abnormal.
- Heart Rate Response to Deep Breathing: Categorized as abnormal if the difference between maximal and minimal heart rates during deep breathing (6 breaths/min) was ≤ 10 beats/min.
- Corrected QT (QTc) interval: Prolongation was determined by a QTc interval > 450 ms for men and > 460 ms for women, calculated using Bazett's formula (QTc = QT/√RR).

The severity of CAN was determined by summing the points assigned for each test result (normal: 0, borderline: 0.5, abnormal: 1). A diagnosis of CAN was established if two or more tests were abnormal or if the total autonomic neuropathy score was ≥ 1.

### 2.4 Statistical Analysis

Descriptive statistics are expressed as means ± standard deviation (SD) for continuous variables and as frequencies (percentages) for categorical variables. Group comparisons between CAN and non‐CAN participants were evaluated using the independent *t*‐test for continuous variables and the Chi‐square or Fisher's exact test for categorical data. Logistic regression analysis was employed to identify factors independently associated with CAN, with adjustments made for confounders including age, sex, duration of diabetes, glycemic control, hypertension, dyslipidemia, smoking, and alcohol consumption. Medication classes were not included as uniform adjustment covariates because they may reflect disease severity and/or act as potential mediators rather than confounders in this cross‐sectional analysis. Therefore, selected medication classes were examined as variables of interest in the multivariable model. Statistical analyses were carried out using standard software (SAS version 9.4; SAS Institute, Cary, NC, USA).

### 2.5 Ethical Consideration

This study received approval from the Institutional Review Board (IRB) of Jeonbuk National University Hospital (IRB no. 2020–01‐016). Owing to the retrospective design of the study and the use of fully anonymized EMR data, the IRB waived the requirement for informed consent.

## 3 Results

### 3.1 Prevalence and Clinical Characteristics of Individuals With and Without CAN

A total of 876 individuals with long‐standing T2DM from eight participating centers were included in the analysis, of whom 766 (88.4%) were diagnosed with CAN. Individuals with CAN were significantly older compared to those without CAN (62.6 ± 9.7 vs. 57.4 ± 9.9 years, *p* < 0.0001) and exhibited a longer duration of diabetes (13.7 ± 4.2 vs. 12.7 ± 4.0 years, *p* = 0.021). While body mass index (BMI), height, and weight did not show significant differences between the groups, the CAN group had a markedly greater prevalence of alcohol consumption (*p* = 0.0136) and a slightly higher proportion of individuals engaged in lifestyle modification, such as exercise (*p* = 0.0297). The prevalence of diabetic nephropathy was higher in those with CAN (15.8% vs. 6.9%, *p* = 0.018), and the frequency of hospitalizations was also increased (1.21 ± 1.33 vs. 0.78 ± 0.75, *p* = 0.012). Regarding medication use, calcium channel blockers (28.3% vs. 14.9%, *p* = 0.004) and aspirin (35.9% vs. 20.8%, *p* = 0.0026) were prescribed more frequently in the CAN group. A summary of these clinical characteristics is presented in Table 1.

**TABLE 1:** Clinical characteristics of Korean individuals with long‐standing T2DM according to the presence of cardiovascular autonomic neuropathy status.

Variable	Without CAN (*n* = 101)	With CAN (*n* = 766)	*p*
Demographic and anthropometric characteristics			
Age (years)	57.35 ± 9.91	62.62 ± 9.66	**< 0.0001**
Sex (male, %)	60 (59.41)	436 (57.07)	0.6553
Height (cm)	163.71 ± 9.22	162.88 ± 9.42	0.4171
Weight	66.02 ± 17.08	67.08 ± 13.48	0.4885
Waist circumference (cm)	83.93 ± 6.29	87.99 ± 8.28	0.2106
BMI	25.31 ± 4.58	25.20 ± 4.15	0.8026
DM duration (years)	12.66 ± 4.01	13.69 ± 4.21	**0.0212**
Smoking (%)	53 (58.89)	456 (62.72)	0.4789
Alcohol drinking (%)	44 (48.35)	451 (61.78)	**0.0136**
Exercise (%)	96 (95.05)	755 (98.56)	0.0297
Laboratory findings			
FPG (mg/dL)	155.25 ± 118.90	145.05 ± 57.11	0.1557
HbA1c (%)	7.98 ± 1.82	7.90 ± 1.73	0.6595
Fasting C‐peptide (ng/mL)	1.86 ± 1.45	2.41 ± 2.13	**0.0216**
PP2 C‐peptide (ng/mL)	2.39 ± 1.27	14.98 ± 170.40	0.7547
Total cholesterol (mg/dL)	166.26 ± 137.79	149.32 ± 39.98	**0.0085**
Triglyceride (mg/dL)	141.88 ± 106.50	146.68 ± 108.10	0.6813
HDL‐C (mg/dL)	49.69 ± 12.59	47.39 ± 12.88	0.1005
LDL‐C (mg/dL)	82.73 ± 33.00	79.32 ± 29.19	0.3239
Creatinine (mg/dL)	0.81 ± 0.20	1.05 ± 3.23	0.4664
Urine albumin (mg/dL)	47.06 ± 225.66	119.30 ± 510.23	0.2007
UACR (mg/g Cr)	89.12 ± 158.07	199.47 ± 810.52	0.2059
Comorbidities and diabetic complications			
Hypertension (%)	69 (68.32)	55 6 (72.58)	0.3688
Hyperlipidemia (%)	81 (80.20)	582 (75.98)	0.3475
CAD (%)	26 (25.74)	242 (31.59)	0.2318
Stroke (%)	2 (1.98)	49 (6.40)	0.0762
PAOD (%)	5 (4.95)	42 (5.48)	0.8242
Diabetic retinopathy (%)	17 (16.83)	128 (16.71)	0.9755
Diabetic nephropathy (%)	7 (6.93)	121 (15.80)	**0.0182**
Diabetic neuropathy (%)	49 (48.51)	408 (53.26)	0.3689
Diabetic foot (%)	1 (0.99)	12 (1.57)	> 0.9999
Hospitalizations (number)	0.77 ± 0.76	1.19 ± 1.32	0.1066
Medication use			
Insulin (%)	27 (26.73)	210 (27.42)	0.885
Metformin (%)	75 (74.26)	570 (74.41)	0.9732
SU (%)	37 (36.63)	334 (43.60)	0.1833
DPP‐4 inhibitors (%)	51 (50.50)	457 (59.66)	0.0788
GLP‐1 RA (%)	1 (0.99)	3 (0.39)	0.3912
SGLT‐2 inhibitors (%)	24 (23.76)	117 (15.27)	**0.0298**
TZD (%)	13 (12.87)	82 (10.70)	0.5124
*α*‐Glucosidase inhibitors (%)	101 (1.00)	19 (2.48)	0.4978
Meglitinide (%)	0 (0.00)	9 (1.17)	0.6088
ACEi (%)	5 (4.95)	57 (7.44)	0.3612
ARB (%)	52 (51.49)	394 (51.44)	0.9926
CCB (%)	15 (14.85)	217 (28.33)	**0.004**
Diuretics (%)	9 (8.91)	121 (15.80)	0.0685
*α*‐Blocker (%)	1 (0.99)	14 (1.83)	> 0.9999
ASA (%)	21 (20.79)	275 (35.90)	**0.0026**
Clopidogrel (%)	10 (9.90)	94 (12.27)	0.4907
Statin (%)	72 (71.29)	531 (69.32)	0.6865
Anti‐depressant (%)	3 (2.97)	11 (1.44)	0.217

*Note:* Continuous variables of independent *t*‐test and categorical variables of Chi (*κ*) square or Fisher's exact test for two different groups were analyzed. Statistically significant values are indicated in Bold (*p* < 0.05). Data are expressed as number (%) or mean ± standard deviation.

Abbreviations: ACEi: angiotensin‐converting enzyme inhibitor; ARB: angiotensin II receptor blocker; ASA: acetylsalicylic acid; BMI: body mass index; CAD: coronary artery disease; CCB: calcium channel blocker; Cr: creatinine; DM: duration diabetes mellitus duration; DM: nephropathy diabetes mellitus nephropathy; DM: neuropathy diabetes mellitus neuropathy; DM: retinopathy diabetes mellitus retinopathy; DPP4: inhibitor dipeptidyl 4 inhibitor; FPG: fasting plasma glucose; GLP1R: agonist glucagon‐like peptide‐1 receptor; HbA1c: glycated hemoglobin A1c; HDL‐C: high‐density lipoprotein cholesterol; LDL‐C: low‐density lipoprotein cholesterol; PAOD: peripheral artery occlusive disease; PP2: C‐peptide 2 h post‐prandial C‐peptide; SGLT2: inhibitor sodium glucose cotransport 2 inhibitor; SU: sulfonylurea; TZD: thiazolidinedione; UACR: urine albumin‐to‐creatinine ratio.

### 3.2 Comparison of Cardiac Autonomic Parameters Between Individuals With and Without CAN

Significant differences in cardiac autonomic parameters were observed between participants with and without CAN. Supine systolic blood pressure was substantially higher in the CAN group compared to the non‐CAN group (124.63 ± 18.33 mmHg vs. 115.19 ± 14.38 mmHg, *p* < 0.0001). In contrast, upright heart rate was significantly lower among individuals with CAN (80.79 ± 12.83 bpm vs. 83.87 ± 10.96 bpm, *p* = 0.0215).

**FIGURE 1:**
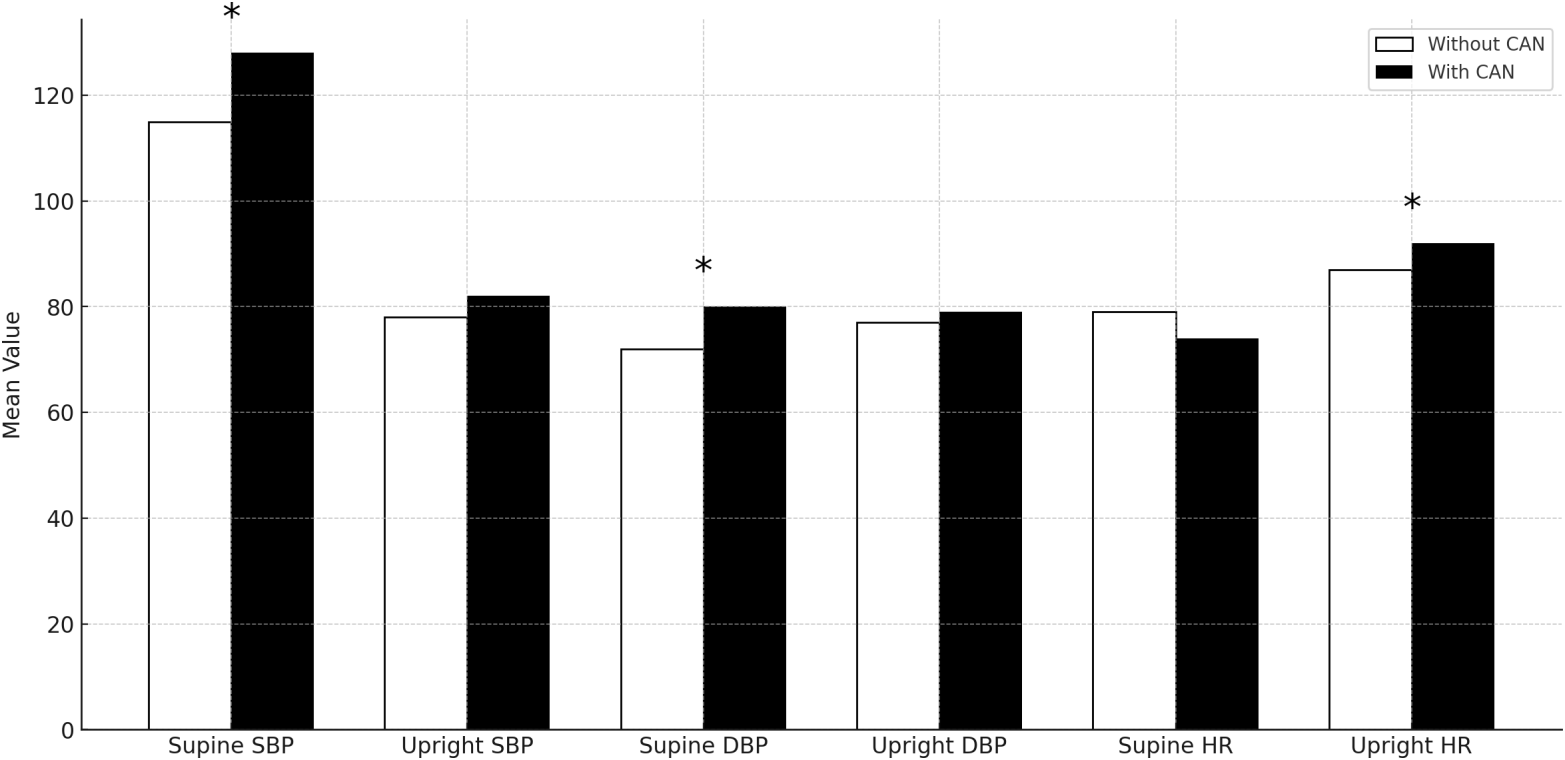
Blood pressure and heart rate differences according to the presence of cardiovascular autonomic neuropathy. Supine systolic blood pressure was significantly higher and upright heart rate significantly lower in individuals with CAN compared to those without CAN. Data are presented as mean ± SD. *p < 0.05 compared with control group. SBP: systolic blood pressure; DBP: diastolic blood pressure; HR: heart rate.

**FIGURE 2:**
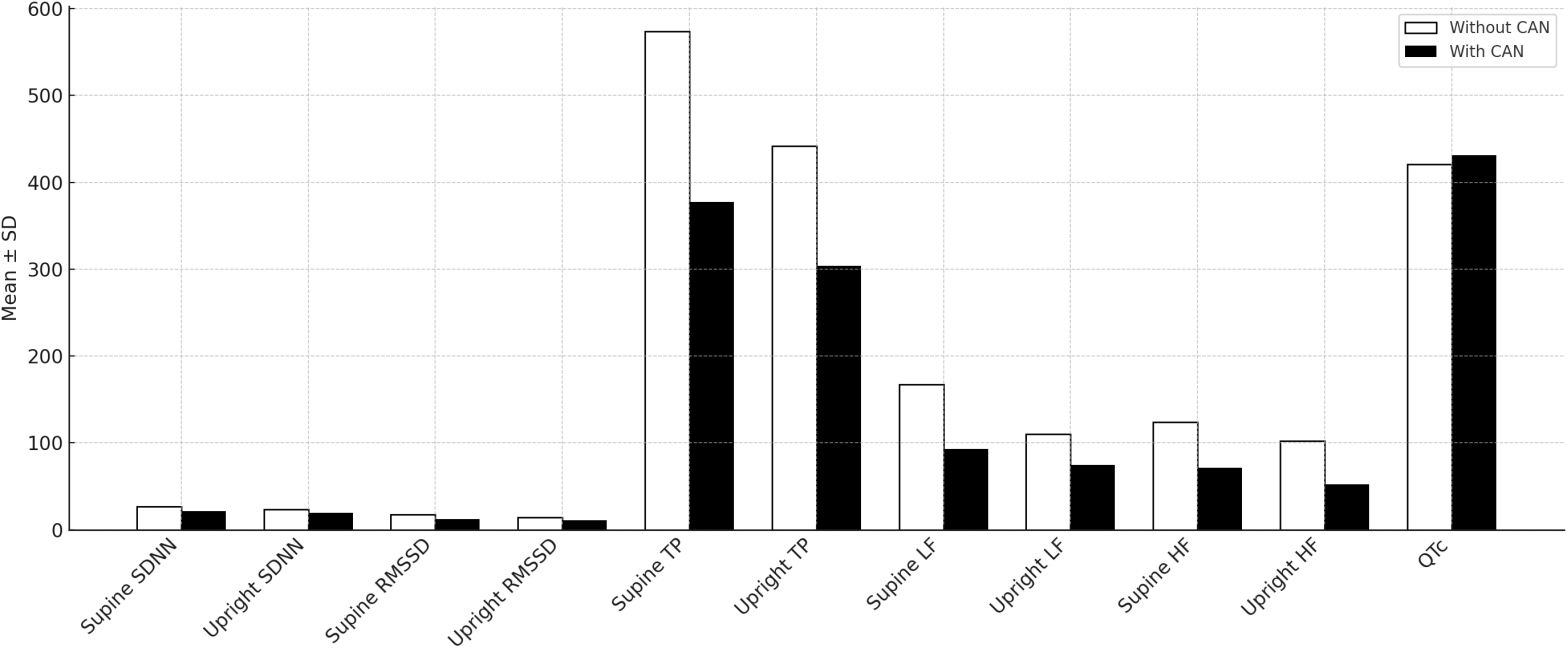
Heart rate variability parameters and corrected QT intervals in individuals with and without cardiovascular autonomic neuropathy. Individuals with CAN exhibited significantly reduced SDNN, RMSSD, TP, LF, and HF components, along with a prolonged QTc interval, compared to those without CAN. Data are presented as mean ± SD. *p* < 0.05 was considered statistically significant. All parameters showed significant differences between groups; therefore, asterisks are not shown. CAN: cardiovascular autonomic neuropathy; SDNN: standard deviation of normal‐to‐normal intervals; RMSSD: root mean square of successive differences; TP: total power; LF: low‐frequency; HF: high‐frequency, QTc: corrected; QT: interval.

Heart rate variability (HRV) indices, including both supine and upright SDNN and RMSSD, were significantly lower in individuals with CAN (all *p* < 0.005). Similarly, total power (TP), low‐frequency (LF), and high‐frequency (HF) components were also decreased in the CAN group in both positions. In contrast, the LF/HF ratio was not significantly different between the groups. Additionally, the corrected QT interval (QTc) on ECG was markedly prolonged in individuals with CAN (431.14 ± 30.07 ms vs. 420.62 ± 18.62 ms, *p* = 0.0006), reflecting more severe autonomic dysfunction. These results underscore the extensive impairment of autonomic regulation in individuals with CAN, as evidenced by abnormal HRV profiles, prolongation of QTc, and altered cardiovascular responses (Table 2, Figures 1 and 2).

**TABLE 2:** Comparison of cardiac autonomic parameters between individuals with and without cardiovascular autonomic neuropathy.

Variable	Without CAN (*n* = 101)	With CAN (*n* = 766)	*p*
Supine SBP, mmHg	115.19 ± 14.38	124.63 ± 18.33	**< 0.0001**
Upright SBP, mmHg	116.97 ± 14.78	122.81 ± 18.53	0.1115
Supine DBP, mmHg	76.06 ± 9.40	77.80 ± 10.40	**0.0024**
Upright DBP, mmHg	79.09 ± 9.91	78.22 ± 10.88	0.444
Supine HR	74.49 ± 9.95	72.56 ± 11.15	0.0984
Upright HR	83.87 ± 10.96	80.79 ± 12.83	**0.0215**
Supine SDNN	26.73 ± 12.34	21.29 ± 11.15	**< 0.0001**
Upright SDNN	23.52 ± 10.35	19.60 ± 10.73	**0.0006**
Supine RMSSD	17.50 ± 17.21	12.37 ± 11.67	**0.0001**
Upright RMSSD	14.57 ± 14.88	11.13 ± 10.92	**0.0046**
Supine TP	573.93 ± 703.94	377.35 ± 529.70	**0.0008**
Upright TP	441.82 ± 420.87	304.00 ± 507.03	**0.0091**
Supine LF, msec^2^	167.40 ± 318.38	93.25 ± 167.54	**0.0003**
Upright LF, msec^2^	109.96 ± 108.70	74.75 ± 177.39	0.0519
Supine HF, msec^2^	124.05 ± 191.71	71.10 ± 123.53	**0.0002**
Upright HF, msec^2^	102.27 ± 203.84	52.29 ± 121.86	**0.0004**
Supine LF/HF ratio	5.09 ± 14.59	4.46 ± 12.75	0.6459
Upright LF/HF ratio	6.19 ± 15.05	5.02 ± 13.80	0.4274
ECG QTc prolongation	420.62 ± 18.62	431.14 ± 30.07	**0.0006**

*Note:* Continuous variables of independent *t*‐test and categorical variables of Chi (*κ*) square or Fisher's exact test for two different groups were analyzed. Statistically significant values are indicated in Bold (*p* < 0.05). Data are expressed as mean ± standard deviation.

Abbreviations: CAN: cardiovascular autonomic neuropathy; DBP: diastolic blood pressure; ECG: electrocardiography; HF: high frequency; HR: heart rate; LF: low frequency; QT: interval; QTc: corrected; RMSSD: root mean square of successive differences; SBP: systolic blood pressure; SDNN: standard deviation of normal‐to‐normal interval; TP: total power.

### 3.3 Factors Associated With CAN in Individuals With Long‐Standing T2DM

Multivariable logistic regression analysis identified several clinical and autonomic factors independently associated with CAN after adjustment for age, sex, diabetes duration, fasting glucose, HbA1c, hypertension, dyslipidemia, smoking, and alcohol consumption.

Among demographic and clinical variables, advancing age was strongly linked to CAN (adjusted OR 1.062, 95% CI, 1.035–1.091; *p* < 0.001). Alcohol consumption was inversely associated with CAN (adjusted OR 0.451; *p* = 0.0238), whereas there were no significant associations for sex, hypertension, or diabetes duration after multivariable adjustment (*p* > 0.2, or inconsistent across models). Although exercise reached statistical significance in the unadjusted model (*p* = 0.0255), this association was not retained following adjustment (*p* = 0.9577).

In metabolic assessments, fasting C‐peptide level emerged as a factor independently associated with CAN (adjusted OR 1.398; *p* = 0.0058), while neither HbA1c nor serum creatinine demonstrated significant associations. Importantly, across all models, HbA1c consistently showed no significant correlation with CAN (adjusted OR range 1.007–1.104; all *p* > 0.2).

With respect to medication exposure, calcium channel blocker (CCB) use had the strongest association with CAN (adjusted OR 4.273; *p* = 0.0002), and use of acetylsalicylic acid (ASA) was also associated (adjusted OR 1.937; *p* = 0.0304). DPP‐4 and SGLT2 inhibitors, however, did not show significant relationships with CAN in adjusted analyses.

Cardiac autonomic measures exhibited strong associations with CAN. Elevated supine and upright SBP were independently associated (adjusted ORs 1.039 and 1.019, respectively), while findings for DBP were less robust, with only supine DBP showing statistical significance (*p* = 0.0024). HRV metrics, including both supine and upright SDNN and RMSSD, were significantly reduced in those with CAN (all *p* < 0.01). Additionally, both LF power and TP in the supine and upright positions were significantly related to CAN. Finally, prolonged QTc on ECG was independently predictive of CAN (adjusted OR 1.017; *p* = 0.0004). A detailed summary of these findings is presented in Table 3.

**TABLE 3:** Multivariable logistic regression analysis of factors associated with cardiovascular autonomic neuropathy.

Variables	With CAN versus without CAN		
	Odd ratio (95% CI)	Unadjusted *p*	Adjusted *p*
Demographic and clinical characteristics			
Age (years)	1.062 (1.035–1.091)	< 0.001	**< 0.001**
Sex	1.64 (0.876–0.899)	0.6553	0.1233
Duration of diabetes (years)	1.026 (0.964–1.092)	0.0212	0.4248
Alcohol consumption	0.761 (0476–1.216)	0.0136	0.2536
Exercise	1.066 (0.101–11.209)	0.0297	0.9577
Smoking (%)	1.515 (0.734–3.126)	0.4789	0.2609
Metabolic parameters and diabetic complications			
FPG (mg/dL)	0.997 (0.993–1.000)	0.1557	0.0593
HbA1c (%)	1.078 (0.935–1.242)	0.6595	0.302
Fasting c‐peptide	1.355 (1.097–1.672)	0.0216	**0.0048**
Serum creatinine	1.944 (0.882–4.285)	0.4664	0.0993
Hypertension (%)	1.228 (0.784–1.922)	0.3688	0.31–0.97
Hyperlipidemia (%)	0.794 (0.449–1.406)	0.3475	0.4288
Diabetic nephropathy (%)	2.272 (0.996–5.182)	0.9577	0.051
Medication use			
DPP4 inhibitor	1.539 (0.954–2.483)	0.0788	0.0771
SGLT2 inhibitor	0.628 (0.355–1.112)	0.0298	0.1104
CCB	4.273 (1.985–9.197)	0.004	**0.0002**
ASA	1.937 (1.065–3.525)	0.0026	**0.0304**
Cardiac autonomic parameters			
Supine SBP, mmHg	1.039 (1.021–1.056)	< 0.001	**< 0.0001**
Upright SBP, mmHg	1.019 (1.004–1.034)	0.1115	**0.0125**
Upright HR	0.988 (0.969–1.007)	0.0215	0.2026
Supine SDNN	0.966 (0.948–0.985)	< 0.0001	**0.0004**
Upright SDNN	0.973 (0.955–0.991)	0.0006	**0.0031**
Supine RMSSD	0.977 (0.960–0.994)	0.0001	**0.0079**
Upright RMSSD	0.973 (0.956–0.991)	0.0046	**0.0028**
Supine TP	1 (0.999–1.000)	0.0008	**0.0156**
Upright TP	1 (0.999–1.000)	0.0091	0.0743
Supine LF, msec2	0.999 (0.998–1.000)	0.0002	0.0265
Upright LF, msec2	0.998 (0.997–0.999)	0.0004	**0.0048**
ECG QTc prolongation	1.017 (1.008–1.027)	0.0006	**0.0004**

Abbreviations: A1c: DPP4 inhibitor dipeptidyl 4 inhibitor; ASA: acetylsalicylic acid; CCB: calcium channel blocker; DBP: diastolic blood pressure; ECG: electrocardiography; HbA1c: glycated hemoglobin; HF: high frequency; HR: heart rate; LF: low frequency; QTc: corrected QT interval; RMSSD: root mean square of successive differences; SBP: systolic blood pressure; SDNN: standard deviation of normal‐to‐normal interval; SGLT2 inhibitor sodium glucose cotransport 2 inhibitor; TP: total power.

## 4 Discussion

CAN is a serious and frequently undiagnosed complication of diabetes mellitus, linked to increased cardiovascular morbidity and mortality [11]. In this multicenter, cross‐sectional study involving Korean patients with long‐standing T2DM, we found a remarkably high prevalence of CAN, affecting approximately 88.4% of participants. This rate is considerably higher than most previously reported prevalence estimates (12%–73%), which mainly reflect differences in study populations and diagnostic methodologies [5, 12]. The elevated prevalence in our cohort is likely due to several contributing factors. First, most participants had experienced diabetes for an extended period which has been consistently associated with the development of CAN. Second, recruitment was limited to secondary and tertiary referral hospitals, where patients generally exhibit more complex or advanced disease. Third, we employed a comprehensive set of autonomic function tests combined with sensitive diagnostic techniques, such as HRV analysis and QTc interval measurement, thereby increasing sensitivity for CAN detection. In 2011, the Toronto Consensus Panel on Diabetic Neuropathy advised the use of cardiovascular reflex tests assessing heart rate and blood pressure changes during specific physiological maneuvers, including heart rate response to deep breathing, standing, the Valsalva maneuver, and BP response to standing [2]. Nevertheless, some recent studies have adopted simplified diagnostic approaches for CAN, applying only a limited selection of CARTs, often utilizing one or two measures such as heart rate response to deep breathing, orthostatic hypotension, or specific HRV frequency domain indices (e.g., SDNN or LF/LH) [12, 13, 14, 15, 16, 17]. Although this abbreviated testing improves feasibility in large‐scale studies, it may result in an underestimation of the true prevalence of CAN, especially in those with subclinical disease.

Although direct statistical comparisons were not conducted, and the lack of a healthy control group is a noted limitation, overall HRV parameters, including SDNN, RMSSD, TP, LF, and HF, were generally lower in our cohort compared to previously published values for age‐matched healthy individuals, except for the LF/HF ratio [18]. A previous meta‐analysis demonstrated that overall HRV parameters are reduced in individuals with T2DM [19], which aligns with the results of our study. Power spectral analyses of heart rate have been shown to be effective not only for the early detection of subclinical CAN but also for diagnosing CAN in patients with advanced chronic complications in whom standard CARTs may be challenging to administer [20]. In addition to HRV parameters, a prolonged QTc interval was identified as an independent factor associated with CAN in our study. Previous research has indicated that a prolonged QTc interval on ECG is not only associated with an increased risk of arrhythmia and sudden cardiac death but also with CAN development [21, 22]. With the recent increase in the use of wearable devices for continuous 24‐h ECG monitoring [23], the integration of these technologies with machine learning approaches is expected to enhance early detection rates of CAN and potentially affect its estimated prevalence.

Consistent with previous reports, advancing age emerged as an independent factor associated with CAN [24]. Conversely, diabetes duration was not significantly associated with CAN in our multivariable analysis. This outcome may be related to the relative homogeneity of our cohort, as only individuals with long‐standing T2DM were included. As a result, diabetes duration may have exhibited insufficient variability to distinguish differences in risk. Likewise, HbA1c was not significantly associated with CAN in our analysis, which contrasts with prior study findings [25]. One explanation may be that participants had relatively stable glycemic control at baseline, as reflected by the narrow range of HbA1c values. In addition, given the progressive and cumulative pathophysiology of CAN, a single baseline HbA1c value may not capture a long‐term glycemic burden or variability relevant to autonomic nerve damage.

Importantly, both supine and upright SBP showed independent associations with the presence of CAN, even after adjustment for hypertension. This finding suggests that actual blood pressure measurements, beyond the binary classification of hypertension status, may better reflect underlying vascular stiffness or autonomic impairment. Previous studies have also demonstrated independent associations between CAN and blood pressure parameters, even in normoalbuminuric patients with T2DM and no prior history of hypertension [26]. In individuals with prolonged diabetes duration, alterations in vascular tone and autonomic regulation may become more pronounced and persistent. Furthermore, in Korea, where stroke contributes substantially to atherosclerotic cardiovascular disease burden, optimizing blood pressure management may provide particular clinical benefit in mitigating adverse outcomes.

Taken together, these findings suggest that CAN in long‐standing T2DM is more closely related to cumulative vascular and autonomic burden than to single metabolic parameters measured at a single time point.

In this context, the potential influence of glucose‐lowering medications on autonomic function warrants careful interpretation. In the present study, SGLT2 inhibitor use was associated with CAN in unadjusted analyses; however, this association was attenuated and did not remain statistically significant after multivariable adjustment. Several factors may explain this finding. First, confounding by indication is likely, because SGLT2 inhibitors tend to be prescribed selectively according to baseline clinical profiles (e.g., age, renal function, and cardiovascular risk), which may also be related to autonomic dysfunction. Second, the multivariable model included blood pressure both as categorical diagnosis (hypertension) and as continuous measures (systolic and diastolic blood pressure). Given the established blood pressure–lowering effects of SGLT2 inhibitors, this covariate structure may have introduced overadjustment and/or collinearity, thereby weakening the apparent independent association of SGLT2 inhibitor use with CAN. Finally, the cross‐sectional design precludes evaluation of treatment duration, cumulative exposure, and dose–response relationships, which may be particularly relevant for autonomic outcomes. Accordingly, the absence of a significant adjusted association should be interpreted cautiously and does not exclude a potential role of STLT2 inhibitors in modulating cardiovascular autonomic function.

Several limitations should be taken into account when interpreting our findings. First, the cross‐sectional design limits the ability to draw causal inferences between the identified associated factors and the occurrence of CAN. Longitudinal studies are warranted to establish temporal relationships and assess the temporal and prognostic significance of the observed associations. Second, despite the use of standardized autonomic function testing, the absence of healthy control subjects restricted direct comparison of HRV parameters and CAN prevalence with the general population. Third, as the study cohort comprised only patients with long‐standing T2DM managed at tertiary referral centers, selection bias likely affected the observed prevalence and clinical features of CAN. Finally, because serial glycemic or blood pressure variability data were not available, we could not evaluate the influence of long‐term glycemic or blood pressure fluctuations on CAN development.

In summary, our study reveals a high prevalence of CAN in individuals with long‐standing T2DM and demonstrates independent associations with advanced age, reduced HRV, prolonged QTc interval, and increased blood pressure. These findings highlight the need for routine integration of autonomic evaluation into diabetes management, with special focus on high‐risk groups. Furthermore, our results indicate that, in addition to standard glycemic control, greater emphasis on cardiac autonomic health and blood pressure regulation may be essential to mitigate diabetes‐related complications. Future longitudinal investigations that utilize wearable devices and continuous physiological monitoring have the potential to refine CAN screening approaches and promote earlier diagnosis.

## Author Contributions

I.B.P. and C.H.K. conceived and designed the study and supervised the research process. J.‐S.Y., J.C.W., J.H.L., and H.‐S.K. contributed to data acquisition, patient recruitment, and clinical investigation at each participating center. S.M.K. and K.H.J. performed the statistical analyses and interpreted the data. T.S.P. provided overall supervision, critically revised the manuscript, and served as the corresponding author. All authors reviewed and approved the final version of the manuscript.

## Funding

The authors have nothing to report.

## Conflicts of Interest

The authors declare no conflicts of interest.

## Data Availability

The data that support the findings of this study are available on request from the corresponding author. The data are not publicly available due to privacy or ethical restrictions.
